# Summarizing Study Characteristics and Diagnostic Performance of Commercially Available Tests for Respiratory Syncytial Virus: A Scoping Literature Review in the COVID-19 Era

**DOI:** 10.1093/jalm/jfac058

**Published:** 2022-07-20

**Authors:** David I Bernstein, Asuncion Mejias, Barbara Rath, Christopher W Woods, Jamie Phillips Deeter

**Affiliations:** Cincinnati Children’s Hospital Medical Center, University of Cincinnati College of Medicine, Cincinnati, OH, USA; Department of Pediatrics, Division of Infectious Diseases, Nationwide Children’s Hospital, The Ohio State University, Columbus, OH, USA; Center for Vaccines and Immunity, Abigail Wexner Research Institute at Nationwide Children’s Hospital, Columbus, OH, USA; Vienna Vaccine Safety Initiative, Berlin, Germany; Université de Bourgogne Franche-Comté, Besançon, France; ESCMID Study Group for Respiratory Viruses (ESGREV), Basel, Switzerland; ESCMID Study Group for Respiratory Viruses (ESGREV), Basel, Switzerland; Infectious Diseases Division, Duke University Medical Center, Durham, NC, USA; ESCMID Study Group for Respiratory Viruses (ESGREV), Basel, Switzerland; Roche Diagnostics Corporation, Indianapolis, IN, USA

**Keywords:** clinical, infectious disease, molecular diagnostics, nucleic-acid-based testing, point-of-care testing systems, viral diseases

## Abstract

**Background:**

Nonpharmaceutical interventions to prevent the spread of coronavirus disease 2019 also decreased the spread of respiratory syncytial virus (RSV) and influenza. Viral diagnostic testing in patients with respiratory tract infections (RTI) is a necessary tool for patient management; therefore, sensitive and specific tests are required. This scoping literature review aimed to summarize the study characteristics of commercially available sample-to-answer RSV tests.

**Content:**

PubMed and Embase were queried for studies reporting on the diagnostic performance of tests for RSV in patients with RTI (published January 2005–January 2021). Information on study design, patient and setting characteristics, and published diagnostic performance of RSV tests were extracted from 77 studies that met predefined inclusion criteria. A literature gap was identified for studies of RSV tests conducted in adult-only populations (5.3% of total subrecords) and in outpatient (7.5%) or household (0.8%) settings. Overall, RSV tests with analytical time >30 min had higher published sensitivity (62.5%–100%) vs RSV tests with analytical time ≤30 min (25.7%–100%); this sensitivity range could be partially attributed to the different modalities (antigen vs molecular) used. Molecular-based rapid RSV tests had higher published sensitivity (66.7%–100%) and specificity (94.3%–100%) than antigen-based RSV tests (sensitivity: 25.7%–100%; specificity:80.3%–100%).

**Summary:**

This scoping review reveals a paucity of literature on studies of RSV tests in specific populations and settings, highlighting the need for further assessments. Considering the implications of these results in the current pandemic landscape, the authors preliminarily suggest adopting molecular-based RSV tests for first-line use in these settings.

**IMPACT STATEMENT**

Viral diagnostic testing in patients with respiratory tract infection is a powerful tool for patient management. This scoping literature review included 77 studies reporting diagnostic performance of commercially available respiratory syncytial virus (RSV) tests (published January 2005–January 2021) and summarized the characteristics of such studies. The collated data suggest that molecular-based RSV tests have higher published sensitivity and specificity than antigen-based tests and thus should be considered for first-line use for timely diagnosis and to detect infections in adults with a low-level viral load. Future studies should investigate the diagnostic performance of RSV tests in adults and in outpatient/household settings.

## Introduction

Respiratory syncytial virus (RSV) infection is responsible for a significant proportion of outpatient visits and hospitalizations in children <5 years old, and is associated with substantial clinical and economic burden (1). Once considered to be a disease of childhood, there is increasing recognition of the prevalence of RSV infection in the community-dwelling (2) and hospitalized adult populations (3–5). Both adult and pediatric patients infected with RSV often present with nonspecific, overlapping symptoms that can lead to difficulty in distinguishing it from influenza, coronavirus disease 2019 (COVID-19) caused by severe acute respiratory syndrome coronavirus 2 (SARS-CoV-2), or other respiratory illnesses (6). Thus, empiric diagnosis is often insufficient and should be supported by viral diagnostic testing to facilitate appropriate treatment, improved surveillance, and timely infection control (7).

Historically, viral culture was the gold standard technique for diagnosis of a productive RSV infection; however, it does not provide timely results to inform clinical management (8). Therefore, real-time reverse transcription-polymerase chain reaction (rRT-PCR), which detects the presence of the virus (active or inactive) with equal or greater sensitivity than viral culture, is often referred to as the reference/gold standard for RSV diagnosis in clinical laboratories (8). There are further modalities available for the detection of RSV with variable diagnostic performance; for example, antigen-based testing is sensitive for detecting RSV in young children but is not sensitive enough for use in older children or adults, as per the CDC guidance, due to lower viral loads in the respiratory specimens of this group (9). Thus, rRT-PCR testing is recommended for adults with suspected RSV infection (9).

Most RSV testing takes place in hospitalized patients (10) where selection bias exists toward more severe cases and pediatric patients, the age group most likely to be hospitalized due to RSV infection (11). Testing for RSV in adults by internists and general practitioners is rare, partially due to lack of awareness (12). A recent international study conducted across 15 countries reported that cases of RSV in adults ≥65 years old were notably underrepresented in national surveillance programs (13). RSV testing is also limited in the younger population as shown by a prospective study in pediatric patients (≤18 years old) in Germany, which revealed that only 8.7% of patients presenting with symptoms of a respiratory tract infection (RTI) underwent viral diagnostic testing during standard-of-care procedures (14). The lack of routine testing for RSV may contribute to the underestimation of disease prevalence, and this has practical implications. In one study based in the emergency department of a US hospital, patients aged 6 to 21 years old accounted for 8.7% of the total number of RSV-positive tests, whereas patients aged 22 to 59 years old, and those aged ≥60 years old accounted for 14.0% and 10.5%, respectively (15). Viral diagnostic testing in pediatric and adult populations helps to tailor patient management and the implementation of hospital infection prevention policies as well as reduce the inappropriate use of antibiotics (16).

Nonpharmaceutical interventions implemented to prevent the spread of SARS-CoV-2 have also impacted the spread of RSV and influenza virus, resulting in a larger population of potential immune-naïve populations, which could lead to an increase in disease burden for future respiratory virus seasons. As a result, models predict sporadic outbreaks and an increase in the prevalence of these diseases (17). Indeed, the CDC recently released a health advisory notice warning of increased interseasonal RSV activity across the southern United States (18), and a similar interseasonal resurgence of RSV has been reported in pediatric populations in another area of the United States (19), Switzerland (20), and Australia (21). Viral diagnostic testing in patients presenting with symptoms of an RTI is a powerful tool for surveillance and patient management during such periods of interseasonal resurgence and for future respiratory virus seasons.

Reducing the time needed to diagnose RSV has been shown to be beneficial in adult and pediatric populations. The duration of time to RSV diagnosis, from the point of hospital admission to test result, is positively correlated with length of hospital stay and antibiotic use in hospitalized adults (10). Additionally, the use of point-of-care (POC) testing for RSV in pediatric patients has been associated with a reduction in the use of antibiotic treatment, the need for further clinical investigations, and time spent in the emergency department (22).

In summary, there is a need to test for RSV in patients with RTIs, which has been enhanced by the current COVID-19 pandemic. There are several commercially available tests for the diagnosis of RSV, but there are no publications that comprehensively discuss the study characteristics and reported diagnostic performance of such tests in patients with acute RTI. As study characteristics have been known to impact diagnostic performance, summarizing the literature will empower healthcare professionals to make decisions about the diagnostic modalities that are optimal for their patient population. The objective of this scoping literature review was to provide a high-level qualitative overview of the literature on commercially available sample-to-answer diagnostic tests for RSV in patients with acute RTI by summarizing the characteristics of published studies, including a preliminary data synthesis on reported diagnostic performance.

## Materials and Methods

### Scoping Review Design, Data Sources, and Search Strategy

This scoping literature review was conducted using the scoping review methodology as described by Peters et al. (23) and the guidance provided in the Preferred Reporting Items for Systematic Reviews and Meta-Analyses Extension for Scoping Reviews (24). PubMed (https://pubmed.ncbi.nlm.nih.gov/) and Embase (https://www.embase.com/) were searched on January 21, 2021 using the search terms and criteria in online Supplemental Table 1 to identify studies reporting on the sensitivity and specificity of commercially available sample-to-answer tests for RSV in patients with acute respiratory infection published between January 2005 and January 2021. Database searches were supplemented by manual searches and references, as appropriate. Duplicate articles and ineligible publication types (narrative reviews, editorials, case reports, addresses, biographies, comments, directories, Festschrifts, interviews, lectures, legal cases, legislation, news, newspaper articles, patient education handouts, popular works) were excluded. The titles and abstracts of the remaining articles were reviewed by 2 independent reviewers in parallel, and discrepancies were resolved through discussion. Full-text articles were then obtained, and a second round of screening was conducted by 2 reviewers working in parallel, with adjudication through discussion. Articles not meeting the inclusion criteria were excluded as necessary.

### Study Selection

This scoping literature review included any peer-reviewed studies in the English language providing original data on the sensitivity and specificity of a commercially available sample-to-answer test for RSV (using any molecular or nonmolecular diagnostic tools) relative to an in-house or commercial rRT-PCR, viral culture, and/or immunofluorescence assay as the reference standard in patients of any age with symptoms of an RTI in any setting. Original research articles, systematic reviews, and meta-analyses were included in the review. Studies that used a noncommercial RSV test, studies where the RSV and reference test were not carried out in the same samples, studies in immunocompromised patients, or studies not otherwise meeting the inclusion criteria were excluded. Health economic analyses and preclinical research studies were also excluded.

### Data Extraction

The following information was extracted, where available: RSV test sensitivity, RSV test specificity, commercial brand of RSV test, data collection (prospective vs retrospective), industry sponsorship, age group of study population (adults were defined as patients ≥18 years old), majority (>75%) specimen type, majority (>75%) setting of patient recruitment, and setting where the RSV test was performed. Any missing data were recorded as “not reported” and included in the data synthesis. The analytical time for each test was taken from its respective manufacturer’s data sheet. Rapid tests, including those that were suitable for use at the POC, were defined as having an analytical time ≤30 min. Where one article reported several relevant sensitivity and specificity values (e.g., when more than one RSV test was studied or if there was a prospective and retrospective arm of the study), then each test or study arm was extracted as a subrecord. Following data extraction, analysis of discordant results between the 2 reviewers was conducted by a third independent party, and discrepancies were resolved through discussion.

### Data Reporting

All data handling was carried out using Microsoft Excel 365 (Microsoft Corporation). The range of sensitivity and specificity values and summary statistics were recorded including the lowest and highest value quoted for a particular RSV test from the relevant subrecords. Sensitivity and specificity ranges were not reported for RSV tests with <3 supporting subrecords.

## Results

### Literature Search Outcome

**Fig. 1.**
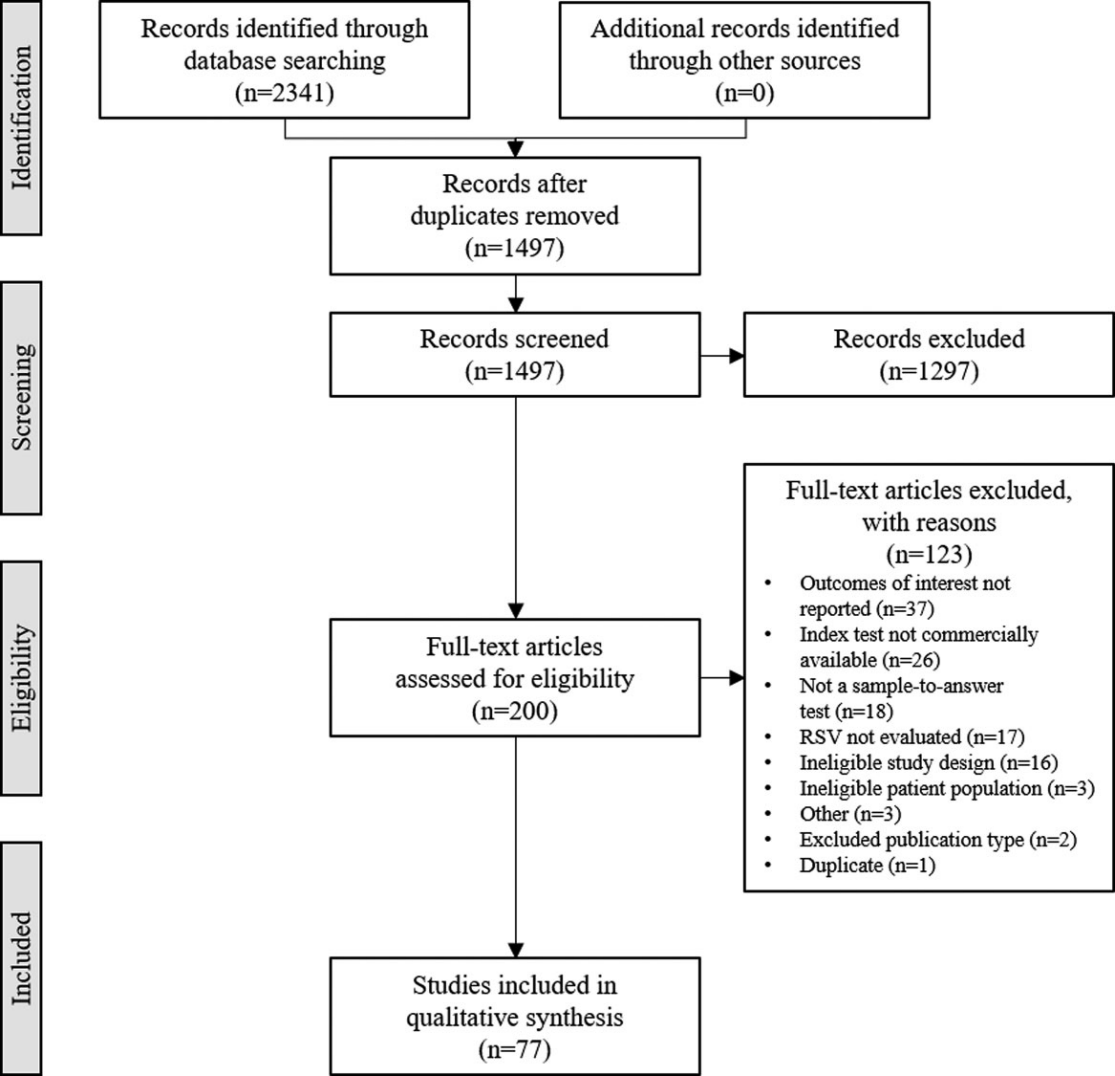
Flow chart summarizing data sources and study selection. Duplicate articles and ineligible publication types were excluded at the screening step (n = 1297). Where 1 article reported several relevant sensitivity and specificity values (e.g., when more than 1 RSV test was studied), then each test was extracted as a subrecord. The 77 included studies corresponded to 133 included subrecords.

Following screening of titles and abstracts, 200 articles were subject to full-text screening, and 77 studies were eventually included in the qualitative synthesis (Fig. 1). In studies reporting several relevant sensitivity and specificity values (e.g., when more than one RSV test was studied), each test was extracted as a subrecord. The 77 included studies corresponded to 133 included subrecords. Overall, the literature search detected 39 different commercially available RSV tests from 27 manufacturers, which represented a variety of technologies and analytical times (Supplemental Table 2).

### Characteristics of All Included Studies and Gap Analysis

**Fig. 2.**
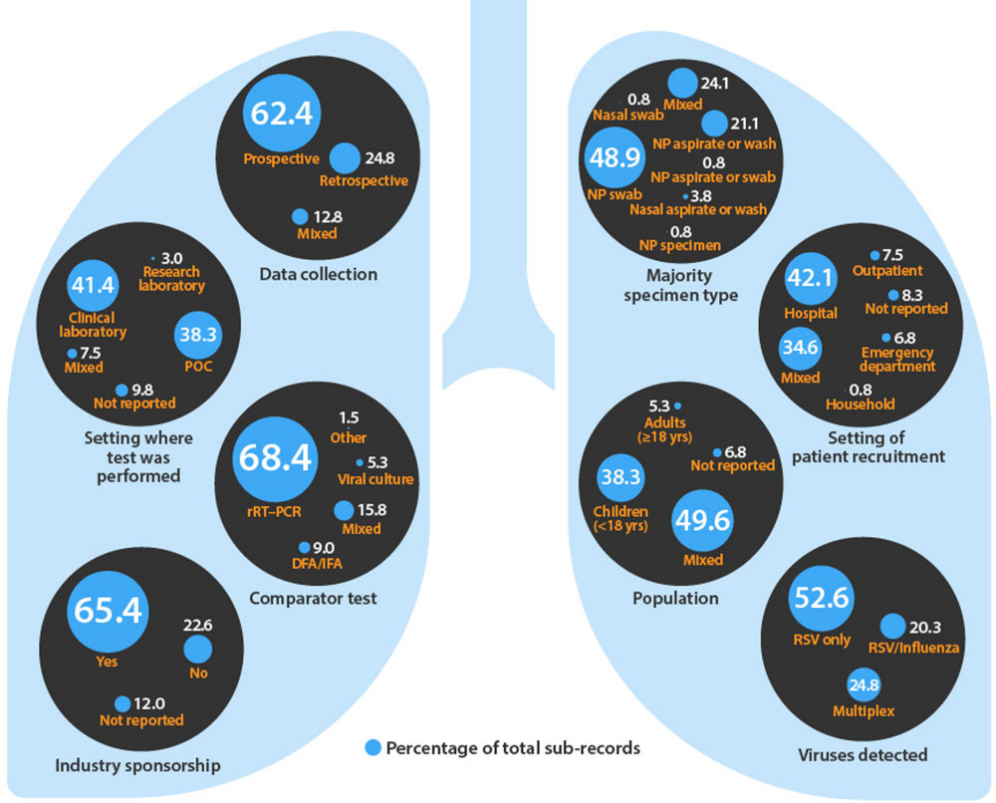
Trends in study design and patient and setting characteristics in studies included in the review. DFA, direct fluorescent antibody test; IFA, immunofluorescence assay; NP, nasopharyngeal.

Most studies examined RSV tests with analytical time ≤30 min relative to RSV tests with analytical time >30 min (66.2% vs 33.8% of included subrecords) (Table 1). The analytical times taken from the manufacturer’s data sheet for each test are shown in Supplemental Table 2. Most studies assessed RSV tests in mixed (49.6% of included subrecords) or pediatric (38.3%) populations, were prospective in design (62.4% of included subrecords), used rRT-PCR as the reference standard (68.4%), and were industry sponsored (65.4%) (Fig. 2). In all studies evaluated, a nasopharyngeal swab was the specimen type most used (48.9% of included subrecords) (Fig. 2). Most patients were recruited when they were admitted to the hospital (42.1%) or from mixed (34.6%) settings (Fig. 2).

**Table 1.** Characteristics of all included studies.

Study characteristic	RSV tests with analytical time ≤30 min, n (%)	RSV tests with analytical time >30 min, n (%)	Total sub-records,^a^ n (%)
Number of subrecords	88 (66.2)	45 (33.8)	133 (100.0)
Commercial index test^b^			
3 M Rapid Detection RSV Test	4 (4.5)	—	4 (3.0)
Aries Flu A/B & RSV Assay	—	4 (8.9)	4 (3.0)
BD Veritor System RSV	9 (10.2)	—	9 (6.8)
Alere BinaxNOW RSV	14 (15.9)	—	14 (10.5)
BioFire FilmArray Respiratory 2.1 Panel	—	4 (8.9)	4 (3.0)
cobas Liat Influenza A/B & RSV Assay	10 (11.4)	—	10 (7.5)
Directigen EZ RSV	8 (9.1)	—	8 (6.0)
ePlex Respiratory Pathogen Panel	—	3 (6.7)	3 (2.3)
ID NOW RSV^c^	5 (5.7)	—	5 (3.8)
mariPOC Respi test	—	4 (8.9)	4 (3.0)
Panther Fusion Flu A/B/RSV Assay	—	7 (15.6)	7 (5.3)
QuickVue RSV	4 (4.5)	—	4 (3.0)
RSV Respi-Strip	3 (3.4)	—	3 (2.3)
Simplexa Flu A/B & RSV	—	3 (6.7)	3 (2.3)
Sofia RSV FIA^d^	11 (12.5)	—	11 (8.3)
Xpert Flu/RSV XC	3 (3.4)	—	3 (2.3)
Xpert Xpress Flu/RSV	6 (6.8)	—	6 (4.5)
Data collection			
Prospective	64 (72.7)	19 (42.2)	83 (62.4)
Retrospective	17 (19.3)	16 (35.6)	33 (24.8)
Mixed	7 (8.0)	10 (22.2)	17 (12.8)
Population			
Adults (≥18 years)	1 (1.1)	6 (13.3)	7 (5.3)
Children (<18 years)	43 (48.9)	8 (17.8)	51 (38.3)
Mixed	41 (46.6)	25 (55.6)	66 (49.6)
Not reported	3 (3.4)	6 (13.3)	9 (6.8)
Comparator test			
DFA/IFA^e^	9 (10.3)	3 (6.7)	12 (9.0)
rRT-PCR	58 (65.9)	33 (73.3)	91 (68.4)
Viral culture	5 (5.7)	2 (4.4)	7 (5.3)
Mixed	16 (18.2)	5 (11.1)	21 (15.8)
Other^f^	0	2 (4.4)	2 (1.5)
Industry sponsorship			
Yes	52 (59.1)	35 (77.8)	87 (65.4)
No	23 (26.1)	7 (15.6)	30 (22.6)
Not reported	13 (14.8)	3 (6.7)	16 (12.0)
Majority specimen type			
Nasopharyngeal aspirate or wash	22 (25.0)	6 (13.3)	28 (21.1)
Nasopharyngeal swab	43 (48.9)	22 (48.9)	65 (48.9)
Nasopharyngeal aspirate or swab	1 (1.1)	0	1 (0.8)
Nasopharyngeal specimen	1 (1.1)	—	1 (0.8)
Nasal aspirate or wash	4 (4.5)	1 (2.2)	5 (3.8)
Nasal swab	1 (1.1)	0	1 (0.8)
Mixed	16 (18.2)	16 (35.6)	32 (24.1)
Setting of patient recruitment			
Emergency department	5 (5.7)	4 (8.9)	9 (6.8)
Hospital	35 (39.8)	21 (46.7)	56 (42.1)
Household	0	1 (2.2)	1 (0.8)
Outpatient	5 (5.7)	5 (11.1)	10 (7.5)
Mixed	37 (42.0)	9 (20.0)	46 (34.6)
Not reported	6 (6.8)	5 (11.1)	11 (8.3)
Setting where test was performed			
Clinical laboratory	26 (29.5)	29 (64.4)	55 (41.4)
POC	46 (52.3)	5 (11.1)	51 (38.3)
Research laboratory	2 (2.3)	2 (4.4)	4 (3.0)
Mixed	10 (11.4)	0	10 (7.5)
Not reported	4 (4.5)	9 (20.0)	13 (9.8)

a Where 1 article reported several relevant sensitivity and specificity values (e.g., when more than 1 index test was studied), then each set of sensitivity and specificity values was extracted as a subrecord; 77 included studies corresponded to 133 included subrecords.

b Only RSV tests with ≥3 subrecords were included in the table. The following index tests had 2 supporting subrecords: CLART PneumoVir, nCounter, NxTAG-Respiratory Pathogen Panel, MultiCode-PLx Respiratory Viral Panel, QIAstat-Dx Respiratory Panel, Simprova-RV, NucliSens EasyQ Respiratory Syncytial Virus A + B assay, Thermo Electron RSV OIA kit, and Verigene Respiratory Virus Plus Nucleic Acid Test. The following index tests had 1 supporting subrecord: Allplex Respiratory Panel 1, Colloidal Gold Genesis, GenRead RSV, Humasis RSV Antigen Test, Magicplex RV Panel Real-Time Test, Prodesse ProFlu+ Assay, RSV K-SeT, Seeplex RV15 OneStep ACE Detection, Bioline RSV, Solana RSV + hMPV, Speed-Oligo RSV, TRU RSV, and Xpect RSV.

c The ID NOW RSV was formerly known as the Alere i RSV.

d Fluorescence immunoassay.

e Direct fluorescent antibody test/immunofluorescence assay.

f “Other” comparator test was the consensus result for all 3 index tests studied (i.e., a ratio of 2:1 positive to negative results was considered a positive result, and vice versa).

For RSV tests with analytical time ≤30 min, most were performed at the POC (52.3%), whereas RSV tests with analytical time >30 min were predominantly conducted in the clinical laboratory (64.4%) (Table 1). Regarding knowledge gaps in the literature, there was a notably small percentage of studies conducted in adult-only patients (5.3%), few conducted in outpatient settings (7.5%), and only one study was conducted in a household setting (Table 1).

### Published Sensitivity and Specificity of Antigen- vs Molecular-Based RSV Tests

RSV tests with analytical time ≤30 min had a greater variability in published sensitivity values (25.7%–100%) (Table 2) relative to RSV tests with analytical time >30 min (62.5%–100%) (Table 2); this is partially reflective of the different assays (antigen and molecular) used for RSV tests with analytical time ≤30 min. The range of published specificity values was similar for RSV tests with analytical time ≤30 min (80.3%–100%) relative to RSV tests with analytical time >30 min (77.0%–100%) (Table 2).

**Table 2.** Published sensitivity and specificity of RSV tests by assay technology in all included studies.

	RSV tests with analytical time ≤30 min (n = 88)		RSV tests with analytical time >30 min (n = 45)
Method	Antigen	Molecular	Molecular
Subrecords, n (%)	61 (69.3)	27 (30.7)	41 (91.1)^a^
Overall			
Sensitivity, %	25.7–100	66.7–100	62.5–100
Specificity, %	80.3–100	94.3–100	77.0–100
Age			
<18 years			
Sensitivity, %	25.7–97.6	84.3–100	74.3–100
Specificity, %	80.3–100	94.3–100	97.8–100
≥18 years^b^			
Sensitivity, %	—	—	62.5–100
Specificity, %	—	—	98.9–100
Mixed age			
Sensitivity, %	57.5–100	77.8–100	63.2–100
Specificity, %	91.8–100	94.7–100	77.0–100
Setting			
Inpatient			
Sensitivity, %	25.7–95.2	98.1–100	80.4–100
Specificity, %	80.3–100	94.3–99.4	91.5–100
Emergency department/outpatient			
Sensitivity, %	67.8–97.6	93–100	62.5–100
Specificity, %	97.6–99.6	96–100	97.8–100

a The mariPOC Respi test (n = 4 subrecords) was included in the RSV tests with analytical time >30 min category as the final result is available after 2 h. The sensitivity and specificity values were not included in the table as there were insufficient data to produce a range of sensitivity or specificity values for relevant patient and setting characteristics.

b Only 1 study assessed an RSV test with analytical time ≤30 min in an adult-only (≥18 years) population; therefore, it was not possible to produce a range of sensitivity or specificity values.

Of RSV tests with analytical time ≤30 min, 70.0% (14/20) were antigen-based, and 30.0% (6/20) were molecular tests (Supplemental Table 2). Overall, molecular RSV tests with analytical time ≤30 min had a higher published sensitivity (66.7%–100%) and specificity (94.3%–100%), relative to antigen-based RSV tests with analytical time ≤30 min (reported sensitivity 25.7%–100% and specificity 80.3%–100%) (Table 2). This trend for higher published diagnostic performance in molecular- vs antigen-based RSV tests with analytical time ≤30 min was preserved in all but one of the categories (specificity in the emergency department/outpatient setting) when the reported sensitivity and specificity ranges were broken down by patient age and setting in which the test was carried out (Table 2).

The published sensitivity values of molecular-based tests was highest for those that detected RSV only (93%–100%), followed by RSV and influenza (66.7%–100%), and then multiplex (≥3 viruses detected) platforms (62.5%–100%) (see Supplemental Table 3). Such summary statistics should be interpreted with caution given the differences in reported sensitivity between different tests with similar modalities and analytical times. For example, the cobas^®^ Liat^®^ Influenza A/B & RSV Assay (Roche Diagnostics International Ltd) and the Xpert Xpress Flu/RSV (Cepheid) are both molecular-based tests that detect RSV and influenza in ≤30 min; however, the sensitivity range reported in the literature for each test is 94.2% to 100.0% and 66.7% to 98.1%, respectively (Supplemental Table 4).

### Published Sensitivity and Specificity of CLIA-Waived Tests for RSV

**Fig. 3.**
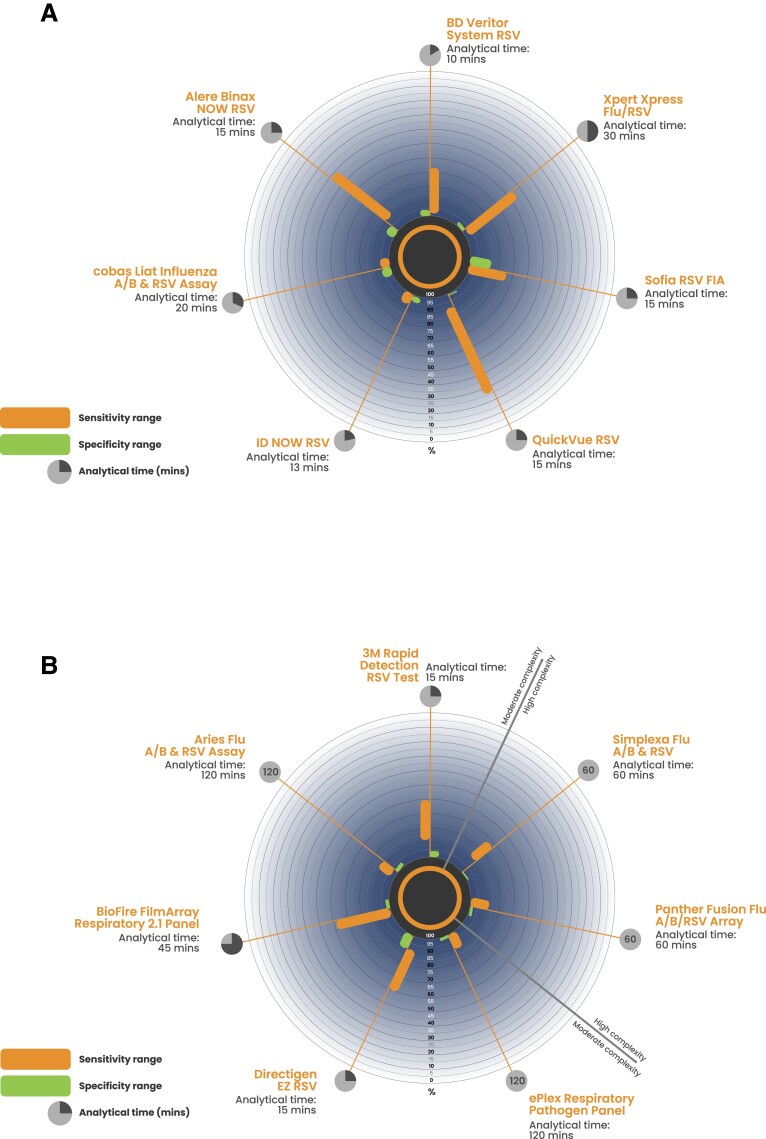
Published sensitivity and specificity of RSV tests under CLIA guidance. The published sensitivity, specificity, and analytical time are shown for the RSV tests included in this review that were CLIA-waived (A) and classed as moderate/high complexity (B). To support interpretation of the diagnostic performance data, the number of studies that were used to extract the published sensitivity and specificity values can be found in Table 3.

There was a variety of published sensitivity and specificity ranges for the 14 RSV tests included in this review that were assessed under CLIA guidance (Fig. 3). Reported sensitivity and specificity values for all RSV tests included in the review are shown in Supplemental Table 4. For the CLIA-waived RSV tests, there was a wide range of published sensitivity (25.7%–100%) and specificity (86.8%–100%) values ( Table 3). The test with the highest range of reported sensitivity values was the cobas Liat Influenza A/B & RSV Assay (94.2% [95% CI, 87.9–97.9] to 100.0% [95% CI, 96.07–100.0]) (Table 3), and the test with the highest reported range of specificity values was the Xpert Xpress Flu/RSV (98.1% [95% CI 96.6–99.0] to 100% [95% CI 99.7–100]) (Table 3).

**Table 3.** Published sensitivity and specificity of RSV tests reviewed under CLIA guidance only.

Test	Manufacturer	Technology	Analytical time, min	Sample type	Age of intended patient population, years	Sensitivity range,^a^ % (95% CI)	Specificity range,^b^ % (95% CI)	Studies, n	References
Waived									
BD Veritor System RSV	Becton, Dickinson and Company	Antigen	10	Nasopharyngeal swab	<6 years	67.5 (56.1–77.6) to 97.6 (NR^c^)	96.8 (91.1–99.3) to 100 (97.0–100)	7	(25–31)
Alere Binax NOW RSV	Abbott	Antigen	15	Nasopharyngeal swab and nasopharyngeal aspirate/wash	<5 years	41.2 (NR) to 90 (NR)	93.2 (92.8–93.6) to 100.0 (97–100)	13	(32–44)
cobas Liat Influenza A/B & RSV Assay	Roche Diagnostics	Molecular	20	Nasopharyngeal swab	Not specified	94.2 (87.9–97.9) to 100.0 (96.07–100.0)	94.29 (86.01–98.42) to 100 (97.7–100)	7	(15, 45–50)
ID NOW RSV^d^	Abbott	Molecular	13	Nasopharyngeal swab	<18 years and ≥60 years	93 (89–96) to 100 (93–100)	96 (93–98) to 98.0 (95.8–99.1)	4	(15, 51–53)
QuickVue RSV	Quidel	Antigen	15	Nasopharyngeal swab and nasopharyngeal aspirate and/or nasal wash	≤18 years	25.7 (NR) to 90.1 (86.8–93.4)	98.5 (NR) to 99.5 (92–99)	4	(30, 54–56)
Sofia RSV FIA^e^	Quidel	Antigen	15	Nasopharyngeal swab and nasopharyngeal aspirate/wash	<19 years	74.8 (68.0–80.9) to 100 (82–100)	86.8 (85.7–87.4) to 100 (95.2–100)	8	(29, 30, 33, 41, 43, 57–59)
Xpert Xpress Flu/RSV	Cepheid	Molecular	30	Nasopharyngeal swab and/or nasal swab	Not specified	66.7 (24.1–94.0) to 98.1 (88.8–99.9)	98.1 (96.6–99.0) to 100 (99.7–100)	5	(45, 60–63)
Moderate complexity									
3M Rapid Detection RSV Test	3M Health Care	Antigen	15	Nasopharyngeal swab and nasopharyngeal aspirate and/or nasal wash	≤21 years	60.0 (38.5–81.5) to 87.3 (83.8–90.1)	95.6 (93.8–96.9) to 99.6 (98.7–100)	2	(64, 65)
Aries Flu A/B & RSV Assay	Luminex Corporation	Molecular	120	Nasopharyngeal swab	Not specified	88.6 (58.3–97.6) to 97.1 (94.4–98.7)	98.4 (97.7–98.9) to 100.0 (98.8–100)	4	(44, 45, 47, 66)
BioFire FilmArray Respiratory 2.1 Panel	bioMérieux	Molecular	45	Nasopharyngeal swab	Not specified	62.5 (24.5–91.5) to 99.4 (96.9–99.9)	98.3 (97.5–98.9) to 100 (97.2–100)	4	(45, 67–69)
Directigen EZ RSV	Becton, Dickinson and Company	Antigen	15	Not specified	<20 years	59 (NR) to 90 (NR)	89.5 (NR) to 99.5 (97.0–100)	6	(30, 32, 42, 70–72)
ePlex Respiratory Pathogen Panel	GenMark Diagnostics	Molecular	120	Nasopharyngeal swab	Not specified	89.6 (80.0–94.8) to 100 (92.6–100)	98.9 (94.2–99.8) to 100 (99.8–100)	2	(73, 74)
High complexity									
Panther Fusion Flu A/B/RSV Assay	Hologic, Inc.	Molecular	Not reported	Nasopharyngeal swab	Not specified	88.4 (81.1–93.1) to 100 (NR)	98.7 (97.0–99.6) to 100 (99.7–100)	6	(45, 47, 74–77)
Simplexa Flu A/B & RSV	Diasorin Molecular	Molecular	60	Nasopharyngeal swab	Not specified	73.3 (44.8–91.0) to 87.0 (74.5–94.2)	99.4 (96.3–99.9) to 100 (98.9–100)	3	(45, 47, 78)

a Sensitivity and specificity ranges are not reported for RSV tests with <3 supporting subrecords.

b Specificity ranges are not reported for RSV tests with <3 supporting subrecords.

c Not reported.

d The ID NOW RSV was formerly known as the Alere i RSV.

e Fluorescence immunoassay.

## Discussion

This scoping literature review summarized the study characteristics and diagnostic performance reported in the peer-reviewed literature for commercially available sample-to-answer tests for RSV. We identified a knowledge gap for studies of RSV tests conducted in adult-only populations or in outpatient or household settings. Overall, RSV tests with analytical time ≤30 min had a greater variability in published sensitivity values relative to RSV tests with analytical time >30 min, which could be partially attributed to the different diagnostic tools (antigen vs molecular) used. Molecular-based rapid RSV tests had higher published sensitivity and specificity ranges than antigen-based RSV tests, which aligns with CDC guidance to use molecular testing for RSV where available (9).

The results from this scoping literature review showed a notable gap in studies of diagnostic performance of RSV tests in adults. Utilizing viral diagnostic testing in adult patients presenting with symptoms of acute respiratory infection would improve current surveillance efforts and allow for efficient triage and treatment decisions (e.g., local infection control guidance could be followed in a timely manner). This could be particularly important for elderly patients who are at a higher risk of hospitalization and death from RSV infection compared with younger adults (79). Testing strategies for SARS-CoV-2 developed in response to the global COVID-19 pandemic have undoubtedly brought diagnostics closer to the patient. With respect to RSV, this review identified few published studies available on the diagnostic performance of tests in outpatient or household settings. The nasopharyngeal shedding of RSV rapidly decreases 1 to 3 days after the onset of symptoms (80); therefore, accessible at-home or POC testing could be a valuable tool in timely infection control.

Approximately half of all studies included in this review used a nasopharyngeal swab as the majority specimen type. Notably, it has been shown that the diagnostic performance of some types of RSV tests is dependent upon sample type. The sensitivity and specificity of immunofluorescence-based RSV tests is higher in nasopharyngeal aspirates, relative to nasal swabs (81, 82). However, there is no difference in test performance between aspirate and swab specimens when using molecular-based RSV testing (81, 83). Additionally, mid-turbinate nasal swabs have been shown to have a comparative viral load to nasopharyngeal swabs in infants <2 years old (84) and are equally sensitive for the diagnosis of multiple respiratory viruses in adults (85). The advantages of using a nasal swab rather than a nasopharyngeal aspirate/swab are that it is less invasive for the patient and easier for clinical staff to transport (81, 83).

The gold standard for RSV testing, rRT-PCR, was the most used comparator assay across all the studies included in this review. The use of different reference standards has been shown to affect the calculated sensitivity and specificity value of an index test; for example, a significant increase in test sensitivity has been reported in the literature when immunofluorescence is used as the reference standard compared with rRT-PCR (32).

A systematic review and meta-analysis of the sensitivity and specificity of RSV rapid antigen-based tests by Chartrand et al. reported a pooled sensitivity and specificity of 80% (95% CI, 76–83) and 97% (95% CI, 96–98), respectively (32). In addition, there was a large disparity observed in sensitivity of RSV tests between studies in pediatric patients (81% [95% CI, 78–84]) and in adults (29% [95% CI, 11–48]). In contrast, a systematic review by Bruning et al. reported that age did not affect diagnostic performance of RSV tests; however, this analysis only focused on 3 rapid RSV tests (BD Veritor System RSV, Sofia RSV FIA, and Alere BinaxNOW RSV) (33). Furthermore, while RSV rapid antigen-based tests are thought to be useful for diagnosis in infants, sensitivity values as low as 7.6% have been reported for a particular brand of rapid antigen-based test in this age group (86).

In some clinical contexts, the use of multiplex tests for more than one respiratory virus may increase efficiency in triaging patients presenting with symptoms of an RTI. Young et al. compared the turnaround time for 2 commercial brands of rapid tests for influenza A and B and RSV. The turnaround time for the ID NOW RSV assay and the ID NOW Influenza A and B assay was 6.4 to 15.8 min per test result vs 21.3 to 22.0 min for the combined cobas Liat Influenza A/B & RSV Assay (87). In addition to considering time to result for multiplex tests, users should also pay close attention to hands-on time when implementing a new assay. Multiplex RSV tests such as the BioFire FilmArray Respiratory 2.1 Panel and the ePlex Respiratory Pathogen Panel, included in this review, can detect >20 infectious respiratory pathogens; however, these tests are not CLIA-waived and have a longer turnaround time but may be extremely valuable in patients with severe disease where rapid identification of the causative agent(s) in a simultaneous manner may be beneficial. In addition, discrepancies in sensitivity between multiplex and RSV-only rRT-PCR tests have been reported in the literature; this could result in varying thresholds for different respiratory viruses between different brands of multiplex rRT-PCR tests (14). The findings from our review also showed that there are differences in the published sensitivity and specificity values for tests for RSV only relative to multiplex tests.

The evidence outlined in this study highlights the need for healthcare professionals to consider the spectrum of respiratory disease, not just SARS-CoV-2 or influenza, and consider how viral diagnostic testing could inform their patient management and treatment decisions. If clinicians do not test for RSV, it leads to selection bias and potentially an underestimation of the prevalence of the virus. Most importantly, it could lead to inappropriate treatment for the patient. Healthcare professionals should assess the benefits and drawbacks of each RSV testing method and decide which would be most appropriate in their practice. Factors to consider include the site of testing, the location of the testing instrument, the age and immune status of the individual being tested, end user of the test, where the test results will be analyzed, the clinical significance of the results, implications for infection control, and the added value of a combination test result (87, 88).

One strength of this scoping review is its comprehensive and structured search strategy, which has maximized the capture of relevant information. In addition, this review has considered a broad spectrum of molecular and nonmolecular RSV tests with different analytical times. The purpose of this scoping review was to summarize the published data available. An inherent limitation of scoping reviews is that the data synthesis is based on the values extracted from any given study; therefore, results may not be comparable in terms of methodology. Any observations related to differences in the published sensitivity and specificity values between RSV tests from different studies should be interpreted with this limitation in mind.

Further research is needed to carry out the statistical analysis required for a full systematic review and/or meta-analysis. The sensitivity and specificity of RSV tests in adult populations and in outpatient and household settings should be assessed. In addition, studies should control for selection bias and adjust for differences in settings where the RSV test was performed, seasonality, and staff utilization of RSV tests. The use of POC testing for influenza and RSV across 4 centers in Denmark resulted in a significant reduction in antibiotic prescription and median hospitalization time in adults (44.3 h) and children (14.2 h); there was also an increase in the use of antiviral treatment in adults only (16). These positive results indicate that further studies are warranted to explore the effects of testing for RSV on patient outcomes (89).

In conclusion, different clinical situations (e.g., the clinical laboratory of a large hospital vs an outpatient clinic) will require different diagnostic solutions. Given the higher published sensitivity and specificity of molecular-based testing over antigen-based modalities for RSV infection, rRT-PCR tests should be considered for first-line use when possible. By summarizing the reported sensitivity and specificity data available in the peer-reviewed literature for commercially available RSV tests, this review might provide an initial reference point for healthcare professionals to further investigate which test is suitable for their practice. Presently, there are several monoclonal antibodies and vaccines in development for RSV prevention and some promising antiviral therapeutic agents for RSV treatment (90). The concurrent use of viral diagnostic testing will be become increasingly important to identify the effectiveness and appropriateness of these products in the future.

## Supplemental Material

Supplemental material is available at *The Journal of Applied Laboratory Medicine* online.

## Supplementary Material

jfac058_Supplementary_Data

## Notes

**Nonstandard Abbreviations:** RSV, respiratory syncytial virus; COVID-19, coronavirus disease 2019; SARS-CoV-2, severe acute respiratory syndrome coronavirus 2; rRT-PCR, real-time reverse transcription-polymerase chain reaction; RTI, respiratory tract infection; POC, point-of-care; FIA, fluorescence immunoassay; NR, not reported.

**Data Availability:** The data supporting this review were derived from publicly available databases.

**Author Declaration:** A version of this article was previously posted as a preprint on medRxiv as https://www.medrxiv.org/content/10.1101/2022.02.14.22270927v1.

**Author Contributions:**  *All authors confirmed they have contributed to the intellectual content of this paper and have met the following 4 requirements: (a) significant contributions to the conception and design, acquisition of data, or analysis and interpretation of data; (b) drafting or revising the article for intellectual content; (c) final approval of the published article; and (d) agreement to be accountable for all aspects of the article thus ensuring that questions related to the accuracy or integrity of any part of the article are appropriately investigated and resolved*.

All authors contributed to the conceptualization of the study, the writing of the original draft and reviewing and editing of the subsequent manuscript drafts. D.I. Bernstein contributed to the formal analysis and methodology of the study. J. Phillips Deeter contributed to the data curation and supervision of the study.

**Authors’ Disclosures or Potential Conflicts of Interest:**  *Upon manuscript submission, all authors completed the author disclosure form. Disclosures and/or potential conflicts of interest:*  **Employment or Leadership:** B. Rath is a member of the ESCMID Study Group for Respiratory Viruses (ESGREV). J. Phillips Deeter is an employee of Roche Diagnostics Corporation and is a member of ESGREV. C.W. Woods is employed by Duke University and Durham VA Health Care System. **Consultant or Advisory Role:** D.I. Bernstein is a paid consultant for Roche Diagnostics. A. Mejias has received fees for participation in advisory boards from Janssen, Sanofi-Pasteur, Merck, and Roche Diagnostics. B. Rath has received fees for participation in advisory boards from Roche Diagnostics and Quidel. C.W. Woods has consultant fees from bioMerieux, Biofire, Giner, Biomeme, FHI Clinical, Arena Pharmaceuticals, SeLux Diagnostics, and Karius, fees from participation in a data safety monitoring board from Janssen, and fees from participation in advisory boards from Regeneron and IDbyDNA. **Stock Ownership:** C.W. Woods holds stocks in Predigen Inc (equity, founder). **Honoraria:** None declared. **Funding:** This review was funded by Roche Diagnostics International Ltd, Rotkreuz, Switzerland. A. Mejias has received research grants from NIH, Janssen, and Merck. B. Rath has received institutional funding support for research from Roche Diagnostics and Quidel. C.W. Woods has received grants from DARPA, NIH/ARLG, NIH/VTEU, and Sanofi and is the principal investigator for RADx UP testing Core. **Expert Testimony:** None declared. **Patents:** C.W. Woods has the following patents: biomarkers for the molecular classification of bacterial infection (issued), methods to diagnose and treat acute respiratory infections (pending), gene expression signatures useful to predict or diagnose sepsis (pending), host based molecular signatures of human infection with SARs-CoV-2 (COVID-19) (pending) and methods of identifying infectious disease and assays for identifying infectious disease (issued).
