# Supplementary material for: Summarizing Study Characteristics and Diagnostic Performance of Commercially Available Tests for Respiratory Syncytial Virus: A Scoping Literature Review in the COVID-19 Era
Source: J Appl Lab Med. 2022 Jul 20:jfac058. doi: 10.1093/jalm/jfac058 (PMC9384538; doi:10.1093/jalm/jfac058)
Supplement: jfac058_Supplementary_Data [file jfac058_supplementary_data.docx]

**SUPPLEMENTAL DATA:**

Summarizing Study Characteristics and Diagnostic Accuracy Performance of Commercially Available Tests for Respiratory Syncytial Virus: A Scoping Literature Review in the COVID-19 Era

| **Supplemental Table 1.** **Data sources and search strategy.** | |
| --- | --- |
| **PubMed, search conducted on January 21, 2021** | |
| **Search number** | **Query** |
| #1 | "Respiratory syncytial virus, Human"[Mesh] OR "Respiratory syncytial virus infections"[Mesh] OR "Bronchiolitis, Viral"[Mesh] OR "respiratory syncytial virus"[tiab] OR bronchiolitis[tiab] OR RSV[tiab] |
| #2 | "Sensitivity"[tiab] OR "Sensitive"[tiab] OR "Specificity"[tiab] OR "Specific"[tiab] OR "Accuracy"[tiab] OR "Accurate"[tiab] |
| #3 | #1 AND #2 |
| #4 | "Immunoassay"[Mesh] OR "Reagent kits, diagnostic"[Mesh] OR "Antigens, viral"[Mesh] OR immunoassay*[tiab] OR immunochromatograph*[tiab] OR "rapid antigen test*"[tiab] OR "rapid antigen detection test*"[tiab] OR "rapid antigen detection"[tiab] OR "antigen detection"[tiab] OR "antigen test*"[tiab] OR "RADT"[tiab] |
| #5 | "Diagnosis"[Majr] OR "Diagnostic Techniques and Procedures"[Majr] OR "Diagnostic Equipment"[Majr] OR "Biological Assay"[Majr] OR "Biological Assay"[Majr] OR "rapid test*"[tiab] OR "rapid detection test*"[tiab] OR "rapid diagnos*"[tiab] OR "rapid detection"[tiab] OR "Quick testing"[tiab] OR "Point of care"[tiab] OR "Point–of–care"[tiab] OR "Bedside testing"[tiab] OR "rapid diagnostic test*"[tiab] |
| #6 | "Nucleic Acids"[Majr] OR "Polymerase Chain Reaction"[Majr] OR "Nucleic acid"[tiab] OR "Nucleic acids"[tiab] OR "PCR"[tiab] OR "Polymerase Chain Reaction"[tiab] OR "Polymerase Chain Reactions"[tiab] OR "RT–PCR"[tiab] OR "Real–time PCR"[tiab] OR "Real time PCR"[tiab] OR "Real–time polymerase chain reaction"[tiab] OR "Real–time polymerase chain reactions"[tiab] OR "Real time polymerase chain reaction"[tiab] OR "Real time polymerase chain reactions"[tiab] OR "Kinetic polymerase chain reaction"[tiab] OR "Kinetic PCR"[tiab] OR "Molecular"[tiab] OR "Molecular test"[tiab] |
| #7 | "Primary Cell Culture"[Majr] OR "Viral culture"[tiab] OR "Tissue culture"[tiab] OR "Culture"[tiab] |
| #8 | #3 AND #4 |
| #9 | #3 AND #5 |
| #10 | #3 AND #6 |
| #11 | #3 AND #7 |
| #12 | #8 OR #9 OR #10 OR #11 |
| #13 | #8 OR #9 OR #10 OR #11 |
| #14 | #8 OR #9 OR #10 OR #11 |
| #15 | #14 NOT ("animals"[Mesh] NOT "humans"[Mesh]) |
| #16 | "Review"[Publication Type] OR "Editorial"[Publication Type] OR "Case Reports" [Publication Type] OR "addresses"[Publication Type] OR "biography"[Publication Type] OR "case reports"[Publication Type] OR "comment"[Publication Type] OR "directory"[Publication Type] OR "festschrift"[Publication Type] OR "interview"[Publication Type] OR "lectures"[Publication Type] OR "legal cases"[Publication Type] OR "legislation"[Publication Type] OR "news"[Publication Type] OR "newspaper article"[Publication Type] OR "patient education handout"[Publication Type] OR "popular works"[Publication Type] OR "case report"[Tiab] OR "report a case"[Tiab] |
| #17 | ((("systematic"[Tiab] OR "systematically"[Tiab]) AND ("review"[Tiab] OR "review"[Tiab] OR "reviewing"[Tiab])) OR "systematic"[sb] OR "Meta–Analysis"[Publication Type] OR "Meta analysis"[Tiab] OR "Meta analyses"[Tiab] OR "Meta–analysis"[Tiab] OR "Meta–analyses"[Tiab] OR "Meta–Analysis as topic"[Mesh]) |
| #18 | #16 NOT #17 |
| #19 | #15 NOT #18 |
| **Embase, search conducted on January 21, 2021** | |
| **Search number** | **Query** |
| #1 | 'human respiratory syncytial virus'/exp OR 'human respiratory syncytial virus' OR 'respiratory syncytial virus infection'/exp OR 'respiratory syncytial virus infection' OR 'viral bronchiolitis'/exp OR 'viral bronchiolitis' OR 'respiratory syncytial virus':ti,ab OR bronchiolitis:ti,ab OR rsv:ti,ab |
| #2 | 'sensitivity':ti,ab OR 'sensitive':ti,ab OR 'specificity':ti,ab OR 'specific':ti,ab OR 'accuracy':ti,ab OR 'accurate':ti,ab |
| #3 | #1 AND #2 |
| #4 | 'immunoassay'/exp OR 'diagnostic kit'/exp OR 'virus antigen'/exp OR immunoassay*:ti,ab OR immunochromatograph*:ti,ab OR 'rapid antigen test*':ti,ab OR 'rapid antigen detection test*':ti,ab OR 'rapid antigen detection':ti,ab OR 'antigen detection':ti,ab OR 'antigen test*':ti,ab OR 'radt':ti,ab |
| #5 | 'diagnosis'/mj OR 'diagnostic procedure'/mj OR 'diagnostic equipment'/mj OR 'bioassay'/mj OR 'rapid test*':ti,ab OR 'rapid detection test*':ti,ab OR 'rapid diagnos*':ti,ab OR 'rapid detection':ti,ab OR 'quick testing':ti,ab OR 'point of care':ti,ab OR 'point–of–care':ti,ab OR 'bedside testing':ti,ab OR 'rapid diagnostic test*':ti,ab |
| #6 | 'nucleic acids, nucleic acid components and their derivatives'/mj OR 'polymerase chain reaction'/mj OR 'nucleic acid':ti,ab OR 'nucleic acids':ti,ab OR 'pcr':ti,ab OR 'polymerase chain reaction':ti,ab OR 'polymerase chain reactions':ti,ab OR 'rt–pcr':ti,ab OR 'real–time pcr':ti,ab OR 'real time pcr':ti,ab OR 'real–time polymerase chain reaction':ti,ab OR 'real–time polymerase chain reactions':ti,ab OR 'real time polymerase chain reaction':ti,ab OR 'real time polymerase chain reactions':ti,ab OR 'kinetic polymerase chain reaction':ti,ab OR 'kinetic pcr':ti,ab OR 'molecular':ti,ab OR 'molecular test':ti,ab |
| #7 | 'primary cell culture'/mj OR 'viral culture':ti,ab OR 'tissue culture':ti,ab OR 'culture':ti,ab |
| #8 | #3 AND #4 |
| #9 | #3 AND #5 |
| #10 | #3 AND #6 |
| #11 | #3 AND #7 |
| #12 | #8 OR #9 OR #10 OR #11 |
| #13 | #12 AND [english]/lim AND [2005–2021]/py |
| #14 | #13 NOT ('animal'/exp NOT 'human'/exp) |
| #15 | #14 AND ('article'/it OR 'article in press'/it OR 'chapter'/it OR 'editorial'/it OR 'erratum'/it OR 'letter'/it OR 'note'/it OR 'review'/it OR 'short survey'/it) |
| #16 | #14 AND ('conference abstract'/it OR 'conference paper'/it) |
| #17 | 'review':it OR 'editorial':it OR 'addresses':it OR 'biography':it OR 'case reports':it OR 'comment':it OR 'directory':it OR 'festschrift':it OR 'interview':it OR 'lectures':it OR 'legal cases':it OR 'legislation':it OR 'news':it OR 'newspaper article':it OR 'patient education handout':it OR 'popular works':it OR 'case report':ti,ab OR 'report a case':ti,ab |
| #18 | ('systematic':ti,ab OR 'systematically':ti,ab) AND ('review':ti,ab OR 'reviewing':ti,ab) OR 'systematic':it OR 'meta–analysis':it OR 'meta analyses':ti,ab OR 'meta–analysis':ti,ab OR 'meta–analyses':ti,ab OR 'meta analysis (topic)'/exp |
| #19 | #17 NOT #16 |
| #20 | #15 NOT #19 |

| **Supplemental Table 2.** **Summary of all commercially available RSV tests included in the literature review.** | | | | | | |
| --- | --- | --- | --- | --- | --- | --- |
| **Assay name** | **Manufacturer** | **CLIA-waived status*^a^*** | **Methodology** | **Technology** | **Virus(es) detected** | **Analytical time**  **(min)** |
| **RSV tests with analytical time ≤30 min** | | | | | | |
| 3M Rapid Detection RSV Test | 3M Health Care | Moderate | Lateral flow chromatographic immunoassay | Antigen | RSV only | 15 |
| Alere BinaxNOW RSV | Abbott | Waived | Lateral flow chromatographic immunoassay | Antigen | RSV only | 15 |
| BD Veritor System RSV | Becton, Dickinson and Company | Waived | Lateral flow chromatographic immunoassay | Antigen | RSV only | 10 |
| Bioline RSV | Abbott | No | Chromatographic immunoassay | Antigen | RSV only | 15 |
| cobas^®^ Liat^®^ Influenza A/B & RSV Assay | Roche Diagnostics | Waived | RT-PCR | Molecular | RSV/Influenza | 20 |
| Colloidal Gold Genesis | Genesis Company | No | Rapid antigen detection test | Antigen | RSV only | Not reported |
| Directigen EZ RSV | Becton, Dickinson and Company | Moderate | Lateral flow chromatographic immunoassay | Antigen | RSV only | 15 |
| GenRead RSV | Orion Diagnostica Oy | No | Reverse transcription strand invasion-based amplification | Molecular | RSV only | Not reported |
| Humasis RSV Antigen Test | Humasis | No | Lateral flow chromatographic immunoassay | Antigen | RSV only | 15 |
| ID NOW RSV*^b^* | Abbott | Waived | Isothermal nucleic acid amplification assay | Molecular | RSV only | 13 |
| QuickVue RSV | Quidel | Waived | Dipstick chromatographic immunoassay | Antigen | RSV only | 15 |
| RSV K-SeT | Coris BioConcept | No | Membrane technology with colloidal gold nanoparticles | Antigen | RSV only | 15 |
| RSV Respi-Strip | Coris BioConcept | No | Dipstick chromatographic immunoassay | Antigen | RSV only | 15 |
| Simprova-RV | Eiken Chemical | No | Loop-mediated isothermal nucleic acid amplification assay | Molecular | RSV/Influenza/ HMPV | 30 |
| Sofia RSV FIA | Quidel | Waived | Antigen detection based lateral flow  immunoassay | Antigen | RSV only | 15 |
| Thermo Electron RSV OIA kit | Thermo BioStar | No | Optical immunoassay | Antigen | RSV only | <20 |
| TRU RSV | Meridian Bioscience | No | Lateral flow chromatographic immunoassay | Antigen | RSV only | 15 |
| Xpect RSV | Thermo Scientific | Waived | Lateral flow chromatographic immunoassay | Antigen | RSV only | 15 |
| Xpert Flu/RSV XC | Cepheid | Moderate | RT-PCR | Molecular | RSV/Influenza | Not reported |
| Xpert Xpress Flu/RSV | Cepheid | Waived | RT-PCR | Molecular | RSV/Influenza | 30 |
| **RSV tests with analytical time >30 min** | | | | | | |
| Allplex Respiratory Panel 1 | Seegene Inc | No | RT-PCR | Molecular | Multiplex | Not reported |
| Aries Flu A/B & RSV Assay | Luminex Corporation | Moderate | RT-PCR | Molecular | RSV/Influenza | 120 |
| BioFire FilmArray Respiratory 2.1 Panel | bioMérieux | Moderate | RT-PCR | Molecular | Multiplex | 45 |
| CLART PneumoVir | Genomica | No | RT-PCR DNA microarray | Molecular | Multiplex | Not reported |
| ePlex Respiratory Pathogen Panel | GenMark Diagnostics | Moderate | RT-PCR | Molecular | Multiplex | 120 |
| Magicplex RV Panel Real-Time Test | Seegene Inc | No | RT-PCR | Molecular | Multiplex | <5 hours |
| mariPOC Respi test | ArcDia | No | Sandwich immunoassay | Antigen | Multiplex | 20–120*^c^* |
| MultiCode-PLx Respiratory Virus Panel | EraGen Biosciences, Inc. | No | RT-PCR | Molecular | Multiplex | Not reported |
| nCounter | NanoString Technologies | No | Digital method of mRNA expression quantification | Molecular | Multiplex | Not reported |
| NucliSens EasyQ Respiratory Syncytial Virus A+B assay | bioMerieux | No | Nucleic acid sequence-based amplification | Molecular | RSV only | <4 hours |
| NxTAG-Respiratory  Pathogen Panel | Luminex Corporation | High | RT-PCR with bead hybridization | Molecular | Multiplex | Not reported |
| Panther Fusion Flu A/B/RSV Assay | Hologic, Inc. | High | RT-PCR | Molecular | RSV/Influenza | Not reported |
| Prodesse ProFlu+ Assay | Hologic, Inc. | High | RT-PCR | Molecular | RSV/Influenza | 4 hours |
| QIAstat-Dx Respiratory Panel | Qiagen | Moderate | RT-PCR | Molecular | Multiplex | 60 |
| Seeplex  RV15 OneStep ACE Detection | Seegene Inc | No | RT-PCR | Molecular | Multiplex | Not reported |
| Simplexa Flu A/B & RSV | Diasorin Molecular | High | RT-PCR | Molecular | RSV/Influenza | 60 |
| Solana RSV + hMPV | Quidel | Moderate | Reverse Transcriptase-Helicase-Dependent Amplification | Molecular | RSV/ HMPV | 45 |
| Speed-Oligo RSV | Vircell | No | RT-PCR | Molecular | RSV only | 90 |
| Verigene Respiratory Virus Plus Nucleic Acid Test | Nanosphere | Moderate | RT-PCR | Molecular | Multiplex | Not reported |
| *^a^*CLIA status is taken from: <https://www.accessdata.fda.gov/scripts/cdrh/cfdocs/cfClia/Search.cfm>, last accessed 04 October 2021. Information regarding the methodology and analytical time of the tests are taken from the respective manufacturers’ data sheets.  *^b^*The ID NOW RSV was formerly known as the Alere i RSV.  *^c^*On average, the mariPOC Respi test reports 80% of positive samples within 20 min and 90% of positive results within 2 hours. The final result within 2 hours reports low positive and negative results.  CLIA, Clinical Laboratory Improvement Amendments; DNA, deoxyribonucleic acid; HMPV, human metapneumovirus; mRNA, messenger ribonucleic acid; RSV, respiratory syncytial virus; RT-PCR, reverse transcription-polymerase chain reaction. | | | | | | |

| **Supplemental** **Table 3. Published sensitivity and specificity of RSV tests by viruses detected in all included studies.** | | | |
| --- | --- | --- | --- |
| **Viruses detected** | **RSV only** | **RSV/Influenza** | **Multiplex** |
| Number of sub-records, n (%) | 70 (52.6) | 27 (20.3) | 33 (24.8)*^a^* |
| **Overall** | | | |
| Sensitivity, % | 25.7–100 | 66.7–100 | 50.0–100 |
| Specificity, % | 77–100 | 94.3–100 | 91.5–100 |
| **Tests using molecular technology only** |  |  |  |
| Sensitivity, % | 93–100 | 66.7–100 | 62.5–100 |
| Specificity, % | 77–100 | 94.3–100 | 91.5–100 |
| *^a^*RSV tests that detected HMPV, Simprova-RV (two sub-records) and Solana RSV + hMPV (one sub-record), were not included in this analysis. HMPV, human metapneumovirus; RSV, respiratory syncytial virus. | | | |

| **Supplemental Table 4.** **Published sensitivity and specificity of all commercially available RSV tests included in the literature review.** | | | |
| --- | --- | --- | --- |
| **Test** | **Sensitivity range, %  (95% CI)** | **Specificity range, %  (95% CI)** | **References** |
| **RSV tests with analytical time ≤30 min** | | | |
| 3M Rapid Detection RSV Test | 60.0 (38.5–81.5)  –  87.3 (83.8–90.1) | 95.6 (93.8–96.9)  –  99.6 (98.7–100) | (1, 2) |
| BD Veritor System RSV | 67.5 (56.1–77.6)  –  97.6 (NR) | 96.8 (91.1–99.3)  –  100 (97.0–100) | (3-10) |
| Binax NOW RSV | 41.2 (NR)  –  90 (NR) | 93.2 (92.8–93.6)  –  100.0 (97–100) | (4, 7, 11-21) |
| cobas Liat Influenza A/B & RSV Assay | 94.2 (87.9–97.9)  –  100.0 (96.07–100.0) | 94.29 (86.01–98.42)  –  100 (97.7–100) | (22-28) |
| Directigen EZ RSV | 59 (NR)  –  90 (NR) | 89.5 (NR)  –  99.5 (97–100) | (9, 12, 19, 29-31) |
| ID NOW RSV*^a^* | 93 (89–96)  –  100 (93–100) | 96 (93–98)  –  98.0 (95.8–99.1) | (27, 32-34) |
| QuickVue RSV | 25.7 (NR)  –  90.1 (86.8–93.4) | 98.5 (NR)  –  99.5 (92–99) | (9, 35-37) |
| RSV Respi-Strip | 36.8 (16.3–61.6)  –  92 (86–96) | 90.2 (83.9–94.7)  –  99 (97–100) | (12, 38, 39) |
| Sofia RSV FIA | 74.8 (68.0–80.9)  –  100 (82–100) | 86.8 (85.7–87.4)  –  100 (95.2–100) | (4, 8, 9, 18, 20, 40-42) |
| Xpert Flu/RSV XC | 90.6 (NR)  –  100 (80–100) | 99.4 (NR)  –  100 (91.9–100) | (43-45) |
| Xpert Xpress Flu/RSV | 66.7 (24.1–94.0)  –  98.1 (88.8–99.9) | 98.1 (96.6–99.0)  –  100 (99.7–100) | (23, 46-49) |
| **RSV tests with analytical time >30 min** | | | |
| Aries Flu A/B & RSV Assay | 88.6 (58.3–97.6)  –  97.1 (94.4–98.7) | 98.4 (97.7–98.9)  –  100.0 (98.8–100) | (21, 23, 24, 50) |
| BioFire FilmArray Respiratory 2.1 Panel | 62.5 (24.5–91.5)  –  99.4 (96.9–99.9) | 98.3 (97.5–98.9)  –  100 (97.2–100) | (23, 51-53) |
| ePlex Respiratory Pathogen Panel | 89.6 (80.0–94.8)  –  100 (92.6–100) | 98.9 (94.2–99.8)  –  100 (99.8–100) | (54, 55) |
| mariPOC Respi test | 50.0 (22.3–77.7)  –  90 (79.5–96.2) | 98.3 (NR)  –  100 (97.5–100) | (56-58) |
| Panther Fusion Flu A/B/RSV Assay | 88.4 (81.1–93.1)  –  100 (NR) | 98.7 (97.0–99.6)  –  100 (99.7–100) | (23, 24, 55, 59-61) |
| Simplexa Flu A/B & RSV | 73.3 (44.8–91.0)  –  87.0 (74.5–94.2) | 99.4 (96.3–99.9)  –  100 (98.9–100) | (23, 24, 62) |
| Sensitivity and specificity ranges were not reported for RSV tests with <3 supporting sub-records. *^a^*The ID NOW RSV was formerly known as the Alere i RSV.  CI, confidence interval; FIA, fluorescence immunoassay; NR, not reported; RSV, respiratory syncytial virus. | | | |

**References**

1. Ginocchio CC, Swierkosz E, McAdam AJ, Marcon M, Storch GA, Valsamakis A, et al. Multicenter study of clinical performance of the 3M Rapid Detection RSV test. J Clin Microbiol 2010;48(7):2337-43.

2. Munjal I, Gialanella P, Goss C, McKitrick JC, Avner JR, Pan Q, et al. Evaluation of the 3M rapid detection test for respiratory syncytial virus (RSV) in children during the early stages of the 2009 RSV season. J Clin Microbiol 2011;49(3):1151-3.

3. Bell JJ, Anderson EJ, Greene WH, Romero JR, Merchant M, Selvarangan R. Multicenter clinical performance evaluation of BD Veritor™ system for rapid detection of respiratory syncytial virus. J Clin Virol 2014;61(1):113-7.

4. Bruning AHL, Leeflang MMG, Vos J, Spijker R, de Jong MD, Wolthers KC, et al. Rapid tests for influenza, respiratory syncytial virus, and other respiratory viruses: a systematic review and meta-analysis. Clin Infect Dis 2017;65(6):1026-32.

5. Cantais A, Mory O, Plat A, Giraud A, Pozzetto B, Pillet S. Analytical performances of the BD Veritor™ System for the detection of respiratory syncytial virus and influenzaviruses A and B when used at bedside in the pediatric emergency department. J Virol Methods 2019;270:66-9.

6. Jonckheere S, Verfaillie C, Boel A, Van Vaerenbergh K, Vanlaere E, Vankeerberghen A, et al. Multicenter evaluation of BD Veritor System and RSV K-SeT for rapid detection of respiratory syncytial virus in a diagnostic laboratory setting. Diagn Microbiol Infect Dis 2015;83(1):37-40.

7. Jung BK, Choi SH, Lee JH, Lee J, Lim CS. Performance evaluation of four rapid antigen tests for the detection of respiratory syncytial virus. J Med Virol 2016;88(10):1720-4.

8. Kanwar N, Hassan F, Nguyen A, Selvarangan R. Head-to-head comparison of the diagnostic accuracies of BD Veritor™ System RSV and Quidel® Sofia® RSV FIA systems for respiratory syncytial virus (RSV) diagnosis. J Clin Virol 2015;65:83-6.

9. Leonardi GP, Wilson AM, Dauz M, Zuretti AR. Evaluation of respiratory syncytial virus (RSV) direct antigen detection assays for use in point-of-care testing. J Virol Methods 2015;213:131-4.

10. Schwartz RH, Selvarangan R, Zissman EN. BD Veritor System respiratory syncytial virus rapid antigen detection test: point-of-care results in primary care pediatric offices compared with reverse transcriptase polymerase chain reaction and viral culture methods. Pediatr Emerg Care 2015;31(12):830-4.

11. Borek AP, Clemens SH, Gaskins VK, Aird DZ, Valsamakis A. Respiratory syncytial virus detection by Remel Xpect, Binax Now RSV, direct immunofluorescent staining, and tissue culture. J Clin Microbiol 2006;44(3):1105-7.

12. Chartrand C, Tremblay N, Renaud C, Papenburg J. Diagnostic accuracy of rapid antigen detection tests for respiratory syncytial virus infection: systematic review and meta-analysis. J Clin Microbiol 2015;53(12):3738-49.

13. Cruz AT, Cazacu AC, Greer JM, Demmler GJ. Performance of a rapid assay (Binax NOW) for detection of respiratory syncytial virus at a children's hospital over a 3-year period. J Clin Microbiol 2007;45(6):1993-5.

14. Khanom AB, Velvin C, Hawrami K, Schutten M, Patel M, Holmes MV, et al. Performance of a nurse-led paediatric point of care service for respiratory syncytial virus testing in secondary care. J Infect 2011;62(1):52-8.

15. Liao RS, Tomalty LL, Majury A, Zoutman DE. Comparison of viral isolation and multiplex real-time reverse transcription-PCR for confirmation of respiratory syncytial virus and influenza virus detection by antigen immunoassays. J Clin Microbiol 2009;47(3):527-32.

16. Miernyk K, Bulkow L, DeByle C, Chikoyak L, Hummel KB, Hennessy T, et al. Performance of a rapid antigen test (Binax NOW® RSV) for diagnosis of respiratory syncytial virus compared with real-time polymerase chain reaction in a pediatric population. J Clin Virol 2011;50(3):240-3.

17. Moesker FM, van Kampen JJA, Aron G, Schutten M, van de Vijver D, Koopmans MPG, et al. Diagnostic performance of influenza viruses and RSV rapid antigen detection tests in children in tertiary care. J Clin Virol 2016;79:12-7.

18. Rack-Hoch AL, Laniado G, Hübner J. Comparison of influenza and RSV diagnostic from nasopharyngeal swabs by rapid fluorescent immunoassay (Sofia system) and rapid bedside testing (BinaxNOW) vs. conventional fluorescent immunoassay in a German university children's hospital. Infection 2017;45(4):529-32.

19. Selvarangan R, Abel D, Hamilton M. Comparison of BD Directigen EZ RSV and Binax NOW RSV tests for rapid detection of respiratory syncytial virus from nasopharyngeal aspirates in a pediatric population. Diagn Microbiol Infect Dis 2008;62(2):157-61.

20. Sun Y, Deng J, Qian Y, Zhu R, Wang F, Tian R, et al. Laboratory evaluation of rapid antigen detection tests for more-sensitive detection of respiratory syncytial virus antigen. Jpn J Infect Dis 2019;72(6):394-8.

21. Voermans JJ, Seven-Deniz S, Fraaij PL, van der Eijk AA, Koopmans MP, Pas SD. Performance evaluation of a rapid molecular diagnostic, MultiCode based, sample-to-answer assay for the simultaneous detection of Influenza A, B and respiratory syncytial viruses. J Clin Virol 2016;85:65-70.

22. Allen AJ, Gonzalez-Ciscar A, Lendrem C, Suklan J, Allen K, Bell A, et al. Diagnostic and economic evaluation of a point-of-care test for respiratory syncytial virus. ERJ Open Res 2020;6(3):00018-2020.

23. Banerjee D, Kanwar N, Hassan F, Essmyer C, Selvarangan R. Comparison of six sample-to-answer influenza A/B and respiratory syncytial virus nucleic acid amplification assays using respiratory specimens from children. J Clin Microbiol 2018;56(11):e00930-18.

24. Banerjee D, Kanwar N, Hassan F, Lankachandra K, Selvarangan R. Comparative analysis of four sample-to-answer influenza A/B and RSV nucleic acid amplification assays using adult respiratory specimens. J Clin Virol 2019;118:9-13.

25. Gibson J, Schechter-Perkins EM, Mitchell P, Mace S, Tian Y, Williams K, et al. Multi-center evaluation of the cobas(®) Liat(®) Influenza A/B & RSV assay for rapid point of care diagnosis. J Clin Virol 2017;95:5-9.

26. Gosert R, Naegele K, Hirsch HH. Comparing the Cobas Liat Influenza A/B and respiratory syncytial virus assay with multiplex nucleic acid testing. J Med Virol 2019;91(4):582-7.

27. Leonardi GP. Evaluation of rapid, molecular-based assays for the detection of respiratory syncytial virus. Intervirology 2019;62(3-4):112-5.

28. Verbakel JY, Matheeussen V, Loens K, Kuijstermans M, Goossens H, Ieven M, et al. Performance and ease of use of a molecular point-of-care test for influenza A/B and RSV in patients presenting to primary care. Eur J Clin Microbiol Infect Dis 2020;39(8):1453-60.

29. Aslanzadeh J, Zheng X, Li H, Tetreault J, Ratkiewicz I, Meng S, et al. Prospective evaluation of rapid antigen tests for diagnosis of respiratory syncytial virus and human metapneumovirus infections. J Clin Microbiol 2008;46(5):1682-5.

30. Vaz-de-Lima LR, Souza MC, Matsumoto T, Hong MA, Salgado MM, Barbosa ML, et al. Performance of indirect immunofluorescence assay, immunochromatography assay and reverse transcription-polymerase chain reaction for detecting human respiratory syncytial virus in nasopharyngeal aspirate samples. Mem Inst Oswaldo Cruz 2008;103(5):463-7.

31. Goodrich JS, Miller MB. Comparison of Cepheid's analyte-specific reagents with BD directigen for detection of respiratory syncytial virus. J Clin Microbiol 2007;45(2):604-6.

32. Schnee SV, Pfeil J, Ihling CM, Tabatabai J, Schnitzler P. Performance of the Alere i RSV assay for point-of-care detection of respiratory syncytial virus in children. BMC Infect Dis 2017;17(1):767.

33. Hassan F, Hays LM, Bonner A, Bradford BJ, Franklin R, Jr., Hendry P, et al. Multicenter clinical evaluation of the Alere i respiratory syncytial virus isothermal nucleic acid amplification assay. J Clin Microbiol 2018;56(3):e01777-17.

34. Peters RM, Schnee SV, Tabatabai J, Schnitzler P, Pfeil J. Evaluation of Alere i RSV for rapid detection of respiratory syncytial virus in children hospitalized with acute respiratory tract infection. J Clin Microbiol 2017;55(4):1032-6.

35. Freeman MC, Haddadin Z, Lawrence L, Piya B, Krishnaswami S, Faouri S, et al. Utility of RSV rapid diagnostic assays in hospitalized children in Amman, Jordan. J Med Virol 2020;93:3420-7.

36. Mesquita FDS, Oliveira DBL, Crema D, Pinez CMN, Colmanetti TC, Thomazelli LM, et al. Rapid antigen detection test for respiratory syncytial virus diagnosis as a diagnostic tool. J Pediatr (Rio J) 2017;93(3):246-52.

37. Rath B, Tief F, Obermeier P, Tuerk E, Karsch K, Muehlhans S, et al. Early detection of influenza A and B infection in infants and children using conventional and fluorescence-based rapid testing. J Clin Virol 2012;55(4):329-33.

38. Gregson D, Lloyd T, Buchan S, Church D. Comparison of the RSV respi-strip with direct fluorescent-antigen detection for diagnosis of respiratory syncytial virus infection in pediatric patients. J Clin Microbiol 2005;43(11):5782-3.

39. Newman H, Tshabalala D, Mabunda S, Nkosi N, Carelson C. Rapid testing for respiratory syncytial virus in a resource-limited paediatric intensive care setting. Afr J Lab Med 2020;9(1):a1084.

40. Gomez S, Prieto C, Folgueira L. A prospective study to assess the diagnostic performance of the Sofia(®) Immunoassay for influenza and RSV detection. J Clin Virol 2016;77:1-4.

41. Tran LC, Tournus C, Dina J, Morello R, Brouard J, Vabret A. SOFIA(®)RSV: prospective laboratory evaluation and implementation of a rapid diagnostic test in a pediatric emergency ward. BMC Infect Dis 2017;17(1):452.

42. Tuttle R, Weick A, Schwarz WS, Chen X, Obermeier P, Seeber L, et al. Evaluation of novel second-generation RSV and influenza rapid tests at the point of care. Diagn Microbiol Infect Dis 2015;81(3):171-6.

43. Popowitch EB, Miller MB. Performance characteristics of Xpert Flu/RSV XC Assay. J Clin Microbiol 2015;53(8):2720-1.

44. Salez N, Nougairede A, Ninove L, Zandotti C, de Lamballerie X, Charrel RN. Prospective and retrospective evaluation of the Cepheid Xpert® Flu/RSV XC assay for rapid detection of influenza A, influenza B, and respiratory syncytial virus. Diagn Microbiol Infect Dis 2015;81(4):256-8.

45. Zelyas N, Shokoples S, Droogers J, Lundeberg R, Leedell D, Drews SJ. Performance of the Alere™ i Influenza A&B and the Cepheid Xpert® Flu/RSV XC assays. Future Virology 2017;12(6):251-9.

46. Cohen DM, Kline J, May LS, Harnett GE, Gibson J, Liang SY, et al. Accurate PCR detection of influenza A/B and respiratory syncytial viruses by use of Cepheid Xpert Flu+RSV Xpress assay in point-of-care settings: comparison to Prodesse ProFlu. J Clin Microbiol 2018;56(2):e01237-17.

47. Haigh J, Cutino-Moguel MT, Wilks M, Welch CA, Melzer M. A service evaluation of simultaneous near-patient testing for influenza, respiratory syncytial virus, Clostridium difficile and norovirus in a UK district general hospital. J Hosp Infect 2019;103(4):441-6.

48. Wabe N, Lindeman R, Post JJ, Rawlinson W, Miao M, Westbrook JI, et al. Cepheid Xpert(®) Flu/RSV and Seegene Allplex(™) RP1 show high diagnostic agreement for the detection of influenza A/B and respiratory syncytial viruses in clinical practice. Influenza Other Respir Viruses 2021;15:245-53.

49. Zou X, Chang K, Wang Y, Li M, Zhang W, Wang C, et al. Comparison of the Cepheid Xpert Xpress Flu/RSV assay and commercial real-time PCR for the detection of influenza A and influenza B in a prospective cohort from China. Int J Infect Dis 2019;80:92-7.

50. Juretschko S, Mahony J, Buller RS, Manji R, Dunbar S, Walker K, et al. Multicenter clinical evaluation of the Luminex Aries Flu A/B & RSV assay for pediatric and adult respiratory tract specimens. J Clin Microbiol 2017;55(8):2431-8.

51. Huang HS, Tsai CL, Chang J, Hsu TC, Lin S, Lee CC. Multiplex PCR system for the rapid diagnosis of respiratory virus infection: systematic review and meta-analysis. Clin Microbiol Infect 2018;24(10):1055-63.

52. Leber AL, Everhart K, Daly JA, Hopper A, Harrington A, Schreckenberger P, et al. Multicenter evaluation of BioFire FilmArray Respiratory Panel 2 for detection of viruses and bacteria in nasopharyngeal swab samples. J Clin Microbiol 2018;56(6):e01945-17.

53. Vos LM, Riezebos-Brilman A, Schuurman R, Hoepelman AIM, Oosterheert JJ. Syndromic sample-to-result PCR testing for respiratory infections in adult patients. Neth J Med 2018;76(6):286-93.

54. Babady NE, England MR, Jurcic Smith KL, He T, Wijetunge DS, Tang YW, et al. Multicenter evaluation of the ePlex Respiratory Pathogen Panel for the detection of viral and bacterial respiratory tract pathogens in nasopharyngeal swabs. J Clin Microbiol 2018;56(2):e01658-17.

55. Sam SS, Caliendo AM, Ingersoll J, Abdul-Ali D, Hill CE, Kraft CS. Evaluation of performance characteristics of Panther Fusion Assays for detection of respiratory viruses from nasopharyngeal and lower respiratory tract specimens. J Clin Microbiol 2018;56(8):e00787-18.

56. Bruning AHL, de Kruijf WB, van Weert H, Willems WLM, de Jong MD, Pajkrt D, et al. Diagnostic performance and clinical feasibility of a point-of-care test for respiratory viral infections in primary health care. Fam Pract 2017;34(5):558-63.

57. Sanbonmatsu-Gámez S, Pérez-Ruiz M, Lara-Oya A, Pedrosa-Corral I, Riazzo-Damas C, Navarro-Marí JM. Analytical performance of the automated multianalyte point-of-care mariPOC® for the detection of respiratory viruses. Diagn Microbiol Infect Dis 2015;83(3):252-6.

58. Tuuminen T, Suomala P, Koskinen JO. Evaluation of the automated multianalyte point-of-care mariPOC® test for the detection of influenza A virus and respiratory syncytial virus. J Med Virol 2013;85(9):1598-601.

59. Pichon M, Valette M, Schuffenecker I, Billaud G, Lina B. Analytical performances of the Panther Fusion System for the detection of respiratory viruses in the French National Reference Centre of Lyon, France. Microorganisms 2020;8(9):1371.

60. Stellrecht KA, Cimino JL, Wilson LI, Maceira VP, Butt SA. Panther Fusion® respiratory virus assays for the detection of influenza and other respiratory viruses. J Clin Virol 2019;121:104204.

61. Voermans JJC, Mulders D, Pas SD, Koopmans MPG, van der Eijk AA, Molenkamp R. Performance evaluation of the Panther Fusion® respiratory tract panel. J Clin Virol 2020;123:104232.

62. Landry ML, Ferguson D. Comparison of Simplexa Flu A/B & RSV PCR with cytospin-immunofluorescence and laboratory-developed TaqMan PCR in predominantly adult hospitalized patients. J Clin Microbiol 2014;52(8):3057-9.
